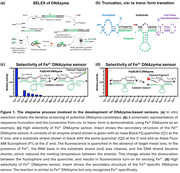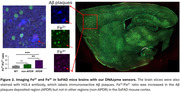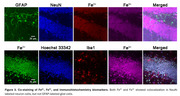# Deciphering Iron Redox Changes in Alzheimer’s Disease using DNAzyme Sensors that can Simultaneously Monitor Fe^2+^ and Fe^3+^

**DOI:** 10.1002/alz.088440

**Published:** 2025-01-03

**Authors:** Yuting Wu, Seyed‐Fakhreddin Torabi, Ryan Lake, Zhenglin Yang, Shanni Hong, Zhengxin Yu, Liviu M Mirica, Laura Fonken, Yi Lu

**Affiliations:** ^1^ the University of Texas at Austin, Austin, TX USA; ^2^ University of Illinois Urbana‐Champaign, Urbana, IL USA

## Abstract

**Background:**

Imbalanced Fe levels can lead to oxidative stress and initiate ferroptosis, an Fe‐dependent cell death that involves lipid peroxidation and can lead to neuron cell loss in neurodegenerative diseases including Alzheimer’s disease (AD). While the Fe^3+^/Fe^2+^ ratio has been identified as the primary determining factor for lipid peroxidation, the role of Fe redox equilibrium and dynamic in AD is not well understood, due to limited tools for visualizing Fe^2+^ and Fe^3+^ simultaneously. To overcome this limitation, we recently reported DNAzyme‐based sensors for simultaneous imaging of Fe^2+^ and Fe^3+^. In this research update, we have integrated the sensors with brain‐wide immunohistochemistry staining to identify cellular correlations between Fe redox changes and AD progression.

**Method:**

We obtained DNAzymes that are highly selective for either Fe^2+^ or Fe^3+^ from a DNA library of up to 10^15^ sequences and used counter‐selection to remove sequences binding competing metal ions. We converted the DNAzymes into fluorescent turn‐on sensors using a method called “catalytic beacon” approach. With these sensors, we imaged Fe^2+^ and Fe^3+^ simultaneously in AD mouse brains. We also performed immunohistochemistry to evaluate neurodegeneration (NeuN), gliosis (Iba1&GFAP), amyloid beta pathology (HJ 3.4), and their correlation with Fe redox changes.

**Result:**

We observed correlated signal changes with the regulation of iron levels. We further applied these sensors in ferroptosis and observed a decrease in Fe^3+^/Fe^2+^ redox ratio over time, indicating Fe redox changes as a potential source of oxidative stress in ferroptosis. These sensors also detected an elevated Fe^3+^/Fe^2+^ ratio in the AD mouse brain, particularly in amyloid plaque regions, suggesting a correlation between amyloid plaques and the accumulation of Fe^3+^ and/or conversion of Fe^2+^ to Fe^3+^. Furthermore, by co‐staining the Fe sensors with immunohistochemistry biomarkers, we found correlations between Fe, Fe redox changes, and neurodegeneration among mice groups differing in genotype, sex, and age.

**Conclusion:**

We have developed highly selective sensors for simultaneously imaging Fe^2+^ and Fe^3+^. By integrating these sensors with immunohistochemistry, we have identified correlations between Fe redox, amyloid plaques, and neurodegeneration in AD mice. Our sensors can offer deep insights into the detailed mechanism of ferroptosis and its role in AD.